# Supplementary material for: Induction of significant neutralizing antibodies against SARS-CoV-2 by a highly attenuated pangolin coronavirus variant with a 104nt deletion at the 3'-UTR
Source: Emerg Microbes Infect. 2022 Dec 18;12(1):2151383. doi: 10.1080/22221751.2022.2151383 (PMC9769135; doi:10.1080/22221751.2022.2151383)
Supplement: Supplemental Material [file TEMI_A_2151383_SM2034.zip › supplementary figures legends_clean version.docx]

**Figure S1.** Viral RNA loads (A) and viral titers (B) in extrapulmonary tissues and blood of GX_P2V(short_3UTR)-infected golden hamsters at different days post-inoculation were determined by qRT-PCR and the TCID_50_ assay, respectively. The detection limit is shown by the dotted line. Error bars represent means±SD.

**Figure S2.** GX_P2V(short_3UTR) caused no histopathological damages in upper respiratory tract and extrapulmonary tissues from intranasally infected golden hamsters. Representative images show that the extrapulmonary tissues from infected golden hamsters had no significant histopathological changes at two days postinfection. Scale bars, 100 μm.

**Figure S3.** Viral RNA loads (A) and viral titers (B) in turbinate, trachea and extrapulmonary tissues of young BALB/c mice intranasally infected with GX_P2V(short_3UTR) were determined by qRT-PCR and TCID_50_ assay, respectively, at one and three days postinfection. The detection limit is shown by the dotted line. Error bars represent means±SD.

**Figure S4.** Intranasal inoculation of GX_P2V(short_3UTR) in young BALB/c mice caused no histopathological damages in extrapulmonary tissues. Representative H&E images of infected tissues from young BALB/c mice at three days postinfection. Scale bars, 100 μm.
