# Supplementary figures and images for: Induction of significant neutralizing antibodies against SARS-CoV-2 by a highly attenuated pangolin coronavirus variant with a 104nt deletion at the 3'-UTR
Source: Emerg Microbes Infect. 2022 Dec 18;12(1):2151383. doi: 10.1080/22221751.2022.2151383 (PMC9769135; doi:10.1080/22221751.2022.2151383)

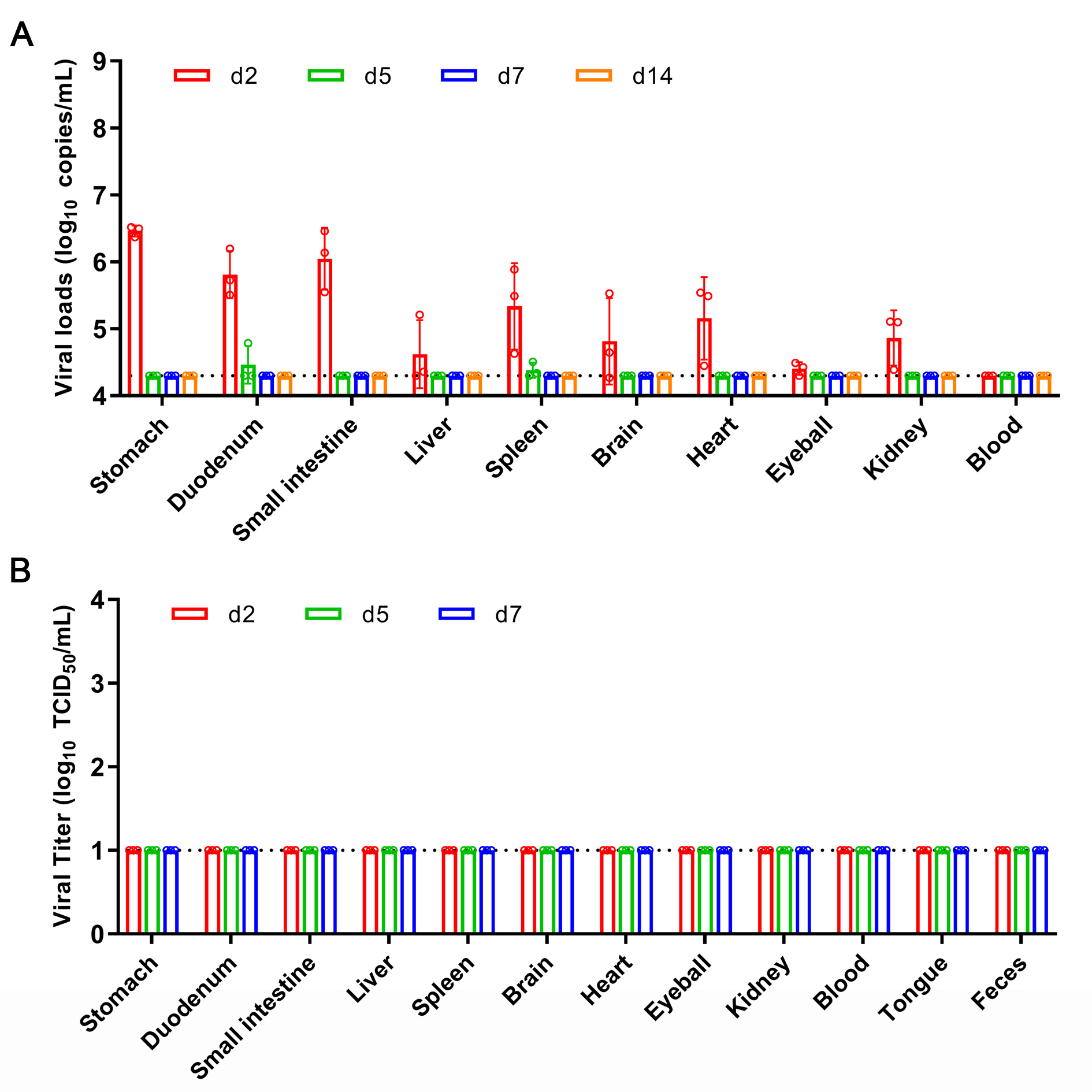

Supplement: Supplemental Material [file TEMI_A_2151383_SM2034.zip › Figure S1.jpg]

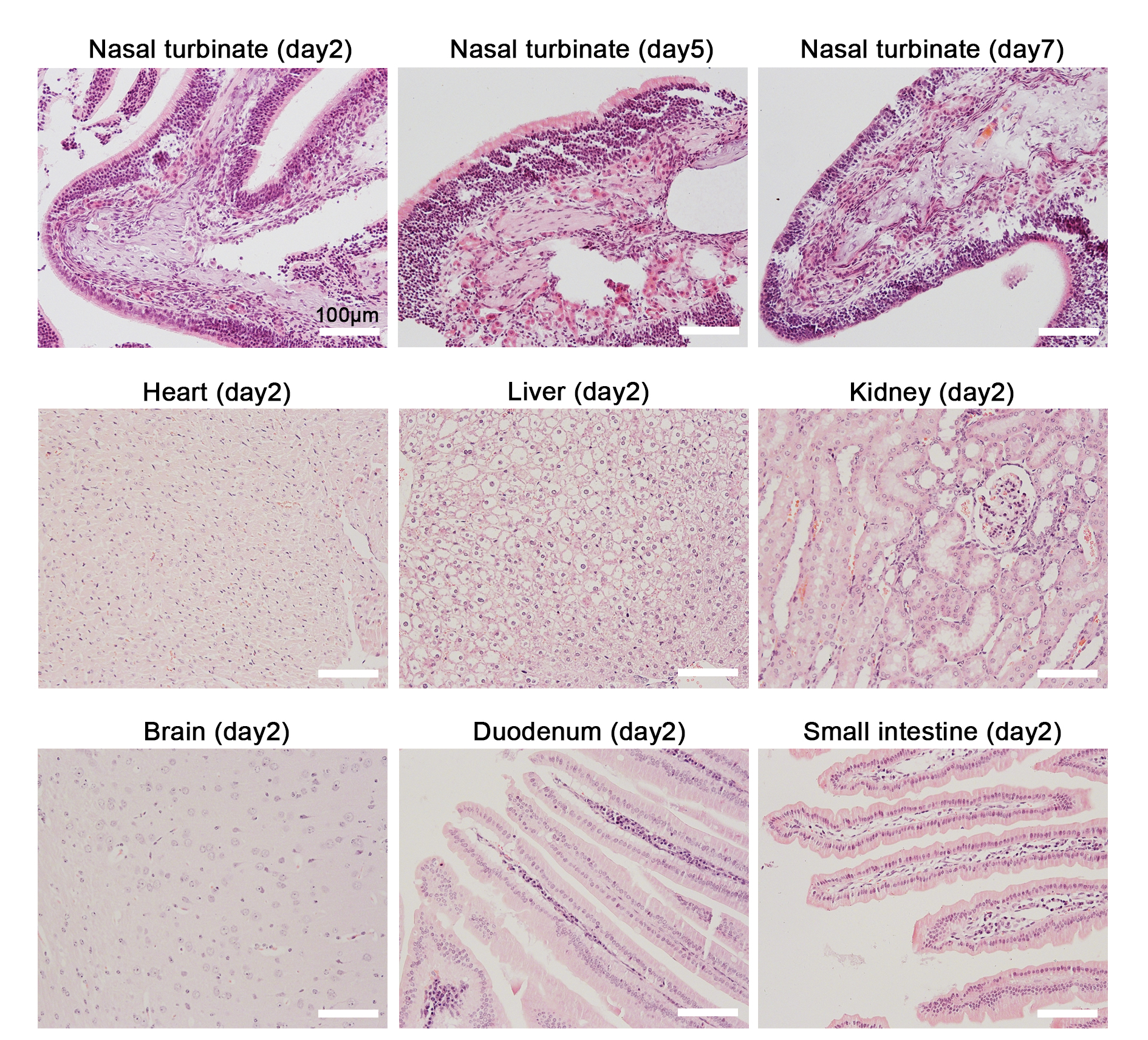

Supplement: Supplemental Material [file TEMI_A_2151383_SM2034.zip › Figure S2.jpg]

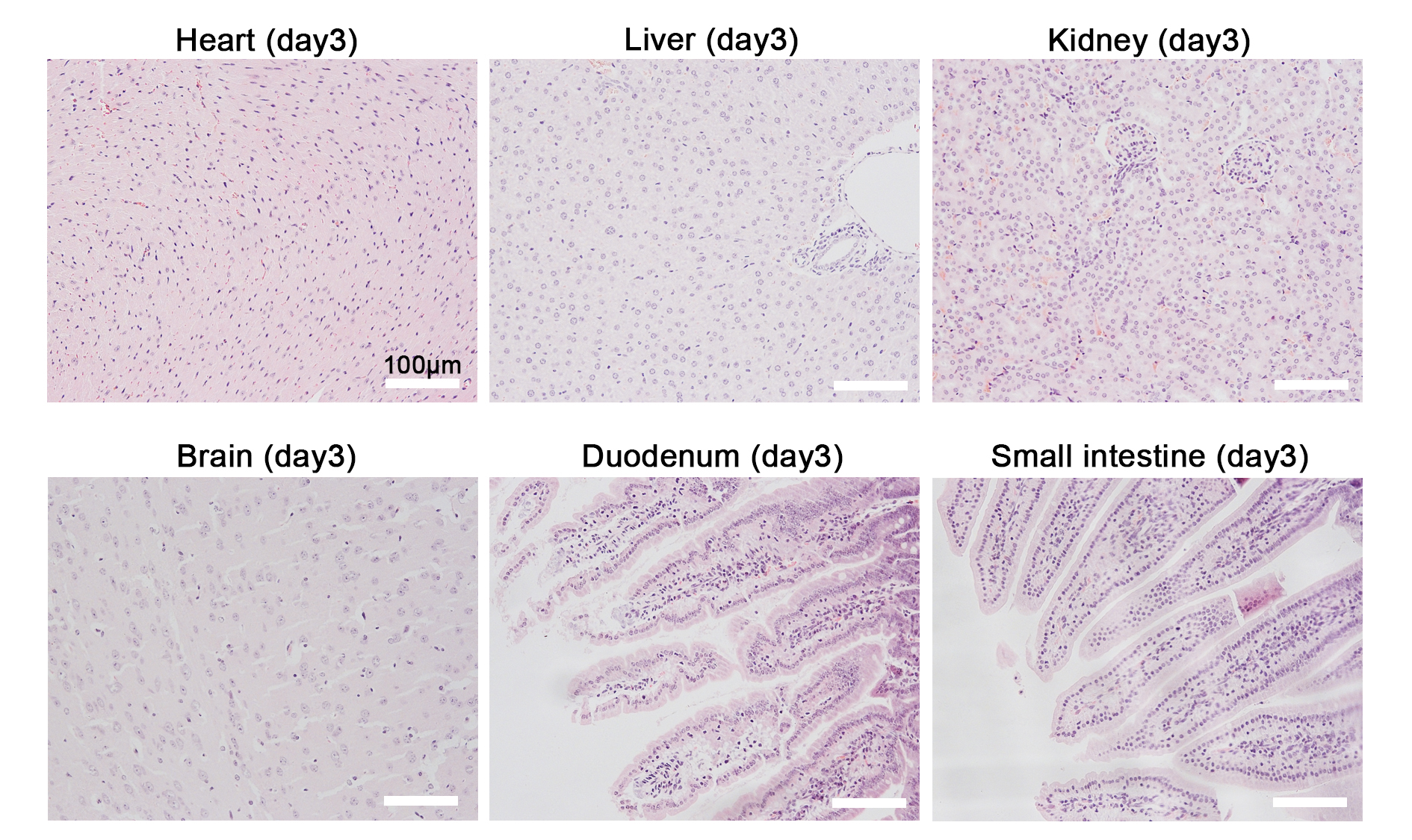

Supplement: Supplemental Material [file TEMI_A_2151383_SM2034.zip › Figure S4.jpg]
